# Oakland's Sugar-Sweetened Beverage Tax: Honoring the “Spirit” of the Ordinance Toward Equitable Implementation

**DOI:** 10.1089/heq.2020.0079

**Published:** 2021-02-02

**Authors:** Yuka Asada, Andrea A. Pipito, Jamie F. Chriqui, Sabira Taher, Lisa M. Powell

**Affiliations:** ^1^ Institute for Health Research and Policy, University of Illinois at Chicago, Chicago, Illinois, USA.; ^2^ Division of Health Policy and Administration, School of Public Health, University of Illinois at Chicago, Chicago, Illinois, USA.; ^3^ Feinberg School of Medicine, Northwestern University, Chicago, Illinois, USA.

**Keywords:** sugar-sweetened beverage tax, health policy, health equity, qualitative research

## Abstract

**Purpose:** On November 8, 2016, Oakland, California, voters passed a sugar-sweetened beverage (SSB) tax, which included language to support programs affecting communities and residents most affected by SSB-related health disparities. The purpose of this study was to qualitatively assess the extent to which those communities most affected by SSB-related health disparities were included in implementation decisions and were recipients of funding to support their needs.

**Methods:** A longitudinal case study from 2016 to 2019 in Oakland, CA, explored equity implementation themes through key informant interview transcripts (*n*=15) triangulated with media (*n*=90) and archived documents (*n*=43). Using principals of constant comparative analysis, all documents (*n*=148) were coded and thematically analyzed in Atlas.ti.

**Results:** SSB taxes—designed to support communities disproportionately impacted by SSB consumption—can be implemented with inclusivity and community representation. The Oakland ordinance established a Community Advisory Board (CAB) that partnered with community organizations throughout implementation to ensure inclusivity and recommend funding for programs to address health inequities, described as the “spirit” of the ordinance. These activities countered the beverage industry's tactics to target lower income communities of color with misinformation campaigns and hinder implementation.

**Conclusion:** A clearly written ordinance provides guidance, which affords an intentional and legal foundation for implementation processes. Establishing a CAB can mitigate inequities as members are invested in the community and initiatives to support residents. Advisory boards are able to liaise between city and local partners, which is a powerful tool for countering opposition campaigns, reaching lower income and communities of color, and ensuring adherence to funding mandates.

## Introduction

Structural inequities in the United States influence access and exposure to foods and beverages associated with chronic diseases and other adverse health risks, such as sugar-sweetened beverages (SSBs). For example, longstanding predatory marketing by the beverage industry has targeted minority communities, increasing SSB desirability, and potentially consumption in these communities.^1–6^ Lower income and non-Hispanic Black households purchased more calories from SSBs than higher income and non-Hispanic White households, respectively.^7^ And, adults with low socioeconomic status (SES), defined by educational attainment and household income, are reported to have higher odds for consuming ≥500 kcal/day from SSBs than high-SES adults.^8^

SSB taxes in the United States have historically faced political resistance despite growing evidence of their potential to decrease SSB consumption, sales, and purchasing.^9–14^ Opponents argue that these taxes are economically regressive, meaning lower income consumers spend a greater share of household income on taxed items than higher income consumers.^15^ Proponents argue that taxes are a progressive public health response because lower income communities of color—who disproportionately suffer from chronic diseases compared with affluent, white counterparts—experience larger health benefits by reducing SSB consumption.^15–17^ Evidence from demand models shows that lower income individuals are more responsive to SSB prices than their higher income counterparts,^18,19^ a response confirmed in recent SSB tax evaluations.^20,21^ Taxes can also attenuate societal inequities, for example, Philadelphia used tax revenues to support the “needs of those at greatest risk of poor health,”^22^ such as a universal pre-K program.^23^ Recently, tax revenues addressed food insecurities exacerbated by the economic impacts of COVID-19.^24,25^

Taken together, it is important to better understand how the implementation of SSB taxes can mitigate the disproportionate rates of chronic diseases experienced by lower income and communities of color. A Berkeley SSB tax assessment noted two key considerations for equitable implementation: appointing an advisory committee with broad capacities, including equity expertise; and, allocating tax revenues that fund “health equity and policy, systems, and environmental change to support chronic disease prevention.”^26^ To our knowledge, no other studies have assessed how SSB tax implementation can address community inequities. However, best practices for equitable interventions have broadly been defined: encouraging inclusivity through leadership and participation from marginalized residents; maintaining cultural sensitivity; placing equal emphasis on roles held by policymakers, researchers, and the community; acknowledging power dynamics by placing equal value on policymaker, researcher, and community roles; and, recognizing and rectifying historical injustices.^27–30^

On November 8, 2016, Oakland, CA, joined five other U.S. cities to adopt an SSB tax, which became effective July 1, 2017.^31^ A Community Advisory Board (CAB), representative of the community (e.g., Oakland Unified School District [OUSD] parents and residents from areas disproportionately impacted by diet-related diseases), was appointed to make tax revenue funding recommendations, “to prevent or reduce health consequences of the consumption of sugar-sweetened beverages…especially those most effected by health disparities.”^31^ The current qualitative study examines equity in the implementation of Measure HH, specifically assessing the extent to which (1) stakeholders and communities most affected by SSB-related health inequities were included in implementation decisions, and (2) those communities received funding to support local community needs. Given the ongoing opposition to SSB taxes in the United States, the ultimate goal of this examination was to inform future activities undertaken by policymakers and advocates working to adopt and implement equitable SSB taxes in their respective cities.

## Methods

This study examined implementation of Oakland's Measure HH. All recruitment, sampling, and data collection and analyses were approved by the Office for the Protection of Research Subjects at the University of Illinois at Chicago (no. 2017-0437).

### Data sources

Three data sources were analyzed for a comprehensive understanding of Measure HH implementation: (1) key informant (KI) interviews; (2) media articles; and (3) archived documents. KIs involved with Measure HH implementation were identified through internet searches and snowball sampling, and were interviewed by telephone at two time points (T1: January to April 2018; T2: January to June 2019). KIs included City of Oakland officials, CAB members, community organization representatives, and a third-party tax administrator. A semistructured interview guide was developed using the Consolidated Framework for Implementation Research domains and settings^32^ and a previously employed interview guide from our team's previous research.^33^ Interviews lasted 45–60 min, were digitally recorded, and professionally transcribed for analysis.

Media articles referencing Measure HH, published January 1, 2016–July 30, 2019 were included and contained fact-based news articles and perspective-based editorials, Op-Eds, and letters to the editor. Databases, including ProQuest, NexusUni, EBSCO, and Google were searched for articles. Nonmedia documents including books, peer-reviewed journals, and gray literature were excluded.

Archived documents published from November 1, 2016 to December 31, 2019 that documented public records, provided a chronology, and furthered context of Measure HH implementation were collected. Process-related materials (e.g., CAB meeting minutes),^34^ official City documentation (e.g., Request for Proposals), and City Department educational and training materials (e.g., information to retailers) were included for review. Complementary materials received from KIs were also included.

### Data analysis

Two trained qualitative researchers coded and analyzed data using first and second pass coding, with an *a priori* coding guide, which was revised continuously until achieving consensus.^35,36^ For the initial coding pass, an inter-rater agreement of >85% was reached before coding commenced on the full data set.^36^ Atlas.ti exploratory functions were applied to explore and organize the data and generate team discussion. Principles of constant comparative analysis were applied during thematic analysis. Themes were primarily derived from interview transcripts; however, documentary evidence review allowed for triangulation and elaboration.

## Results

### Participant characteristics

Table 1 provides a detailed sample description by data source. Across T1 and T2, 15 KI interviews were conducted; documentary evidence searches yielded 90 media and 43 archived documents. In total, 148 documents were included for analysis.

**Table 1.** Sample Characteristics Across 2016–2019

Source by type of data collection	Documents	
	*n*	% within source type^a^
Key informant interview transcripts		
City of Oakland Officials (Finance and Human Services Departments)	5	33
CAB members	2	13
Community advocates/representatives	6	40
Third party tax administration agents	2	13
Sub-total	15	100
Archived documents		
CAB meeting minutes	25	58
Ordinances and regulations	2	5
Request for proposals/quotes	5	12
Presentations and education materials	11	26
Subtotal	43	100
Media articles		
News articles	73	81
Editorials	3	3
Op-Eds	9	10
Letters to the editor	5	5
Subtotal	90	100
Total	149	100

^a^ % column (subtotals) may not sum to 100% owing to rounding.

CAB, Community Advisory Board.

The foundation for Measure HH was centered around addressing inequities using community representation, described by stakeholders as the “spirit” of the ordinance. The CAB was a critical structure that preserved this intention and their dedication to implementation activities promoting inclusivity and partnerships countered the ongoing attacks by the beverage industry to target lower income communities of color.

### Critical role of the CAB in helping to ensure inclusivity and targeted funding

Following the example of Berkeley, CA, Oakland's Measure HH intentionally required the formation of CAB that represented Oakland's diverse communities. From the onset of the CAB's establishment, community members and local advocacy organizations were engaged and assisted in identifying local structural inequities. One community advocate described the CAB:

*So we knew from the start that this was a very receptive board* [CAB]*…meaning that they wanted to see that the funds were targeted…to neighborhoods that had been disproportionately affected by sugar-sweetened beverages…They also wanted to make sure that the monies that go through the projects really addresses not just kind of the health impacts, but looks at the root causes of why these conditions impact low-income communities of color disproportionately.*—Community Advocate

With this strong commitment to preserve the “spirit” of the ordinance, the CAB played a strong role to ensure (1) inclusivity and community representation; (2) partnerships to obtain input directly from the communities most marginalized by structural health inequities; and finally (3) funding recommendations that benefited communities most in need. Each subtheme is described next.

#### Inclusivity and community representation

The CAB, which included local experts, relied on existing professional partnerships to build a network of community stakeholders with a shared commitment to reducing health and social inequities. Once existing partners were enlisted, they engaged their partners, creating a sequence of connections, which amplified access to resources and community reach. For example, the Oakland Food Policy Council actively encouraged community organizations to attend and participate at CAB meetings.^34^ As one local community advocate reflected:

*Thirteen community-based organizations actually showed up to speak on the soda tax. But that wouldn't have had happened…without the work of Oakland Food Policy Council and the Sugar Freedom Project and those of us that have been following up on the implementation.*—Community Advocate

Although the CAB provided recommendations to the City Council, final approval or rejection was not in the CAB's control. However, the cadre of CAB- orchestrated community voices generated a collective power, which was used to influence decision making in the absence of legal authority. Furthermore, the CAB could “highlight and center equity” through implementation, as intended, empowering the community.

*…Never ever take your eye off the ball…the CAB is still an advisory body. The power still resides in the elected officials…One has to have a strategy that will include exercising influence, not just with the advisory board, but with the municipality…Because generally what happens is once you have the policy done and the framework of the implementation set, which is what we've been able to do, and work with the advisory board to get that framework as to highlight and center equity, which is what we wanted, which was what the ordinance prescribed and what we wanted to see happen.*—Community Advocate

#### Partnerships to reach marginalized communities

The CAB leveraged community partnerships, however, two local organizations, the Oakland Food Policy Council and Sugar Freedom Project, were instrumental to their work. The Oakland Food Policy Council is a 21-seat council that advocates for an equitable and sustainable food system.^37^ The Sugar Freedom Project was funded by the California Endowment with a mandate to address diabetes, obesity, “corporate sugar's ubiquity, and the soda tax” in Oakland's lower income communities of color.^38^ This collaboration provided timely access, insight, and community-tailored feedback, a process that avoided bureaucratic delays and hurdles associated with acquiring information through City channels. For example, in a landscape analysis, the City Human Services Department interviewed 20 residents, whereas CAB partners—relying on volunteers and existing networks—were able to survey over 600 residents.^34^ The latter gathered information on SSB consumption across Oakland neighborhoods, locations of soda outlets (e.g., corner stores), and perceptions of how soda consumptions impacts residents.^34^

*And so because Oakland Food Policy Council has the flexibility and is not a bureaucracy in the way that the City of Oakland is, we literally can take that on very easily and just get that information back to them* [CAB]*…and because of priorities and the reach that we have with other community organizations who have access to information.*—Community Advocate

#### Equitable allocation of tax revenues

The City Council approved CAB recommendations funded a wide range of programs, many targeting communities disproportionately affected by SSB consumption. For example, the first recommendation was for “Quick Win” funding—defined as a quick response to an urgent and high-risk community need—that installed water filtration systems to OUSD and Head Start centers, combatting health risks associated with high lead levels reported in a small number of schools.^34^ The CAB also recommended funding for existing Oakland programs that service lower income communities of color, such as the OUSD Food Program and Parks and Recreation.^34^

*“We have a goal for every kid in Oakland to learn to swim by 5th grade, and we have money to provide scholarships for that in the Parks & Rec budget,” he* [Camp Representative] *said. With 26% of children living below the poverty line in Oakland as of 2017 census data, that could go a long way toward mitigating health and fitness disparities among lower-income communities of color.*—*East Bay Express, 2019*^39^

Finally, the addition of the Community Grants Program allocated tax revenues to support local community organizations, targeting lower income priority populations (e.g., West and East Oakland).^34^ While stakeholders described initial City engagement challenges during the Request for Proposals process, the grants program has accounted for 40% of available funding in 2018–19, 2019–20, and 2020–21 and reached a variety of local organizations.^34^ For FY 2019–20, the program funded 14 organizations, totaling ∼$2 million in four key areas: public health, health care, policy, and advocacy.^34^ Table 2 lists examples of program recipients, initiatives, and funding amounts. Additional funding information for equity programs can be found elsewhere.^40^

**Table 2.** Examples of Recommended Community Grants Program Funded Initiatives, Organizations and Funding Amounts (2019–20)

Funding area and example target initiatives	Example organization	Program description and funding amount
Prevention through education and promotion (e.g., increasing consumption of healthy foods, water)	Planting justice	Provide sustainable agriculture, garden training and education to choose healthier food; $150,000
Healthy neighborhoods and places (e.g., neighborhood initiatives to increase access to healthy and affordable food and active living)	Native American Health Center, Inc.	Provide nutrition and wellness education opportunities, Food Farmacy and Food as Medicine health fair programs; $150,000
Health care prevention and migration **(**e.g., prevention of chronic disease to reduce disparities)	Healthy Options at Point of Sale	Engage transitional-aged youth in a community action research project to determine the prevalence of and attitudes toward SSBs; $149,997

Source: Oakland SSB Tax CAB Meeting Minutes.

SSB, sugar-sweetened beverage.

### Beverage industry intentionally targeted lower income communities of color

Consistent with opposition initiatives designed to stifle taxes across the U.S.,^41^ the “No Oakland Grocery Tax” campaign was established with support from the political action committee of the American Beverage Association. The campaign launched before November 2016 and continued through implementation spreading misinformation—that the tax would be applied as an overall “grocery tax,” to increase prices of staples such as bread and milk—particularly in lower-income communities of color. Flyers were also distributed in communities of color, as observed by Oakland Food Policy Council:

*So, we were trying to resist the misinformation as a council* [Oakland Food Policy Council], *that was being put out to specifically communities of color and in Oakland that's mostly East Oakland and West Oakland. And I live in East Oakland, so I would get a flyer saying, “Vote No” on the grocery tax. I probably got one 4 days a week for a good 3 months…That was the biggest push was trying to actually give community folks and voters accurate information that this wasn't a grocery tax.*—Community Advocate

Additionally, the misinformation campaign sympathized with lower-income communities of color by promoting the narrative of tax regressivity (e.g., that the tax would “hurt these communities the most.”)^42^

*These kinds of regressive taxes are not supported by the people of California because they place an unfair burden on working families and neighborhood businesses already struggling with the state's high cost of living*.—February 2019, American Beverage Association representative on CNBC^43^

**FIG. 1.**
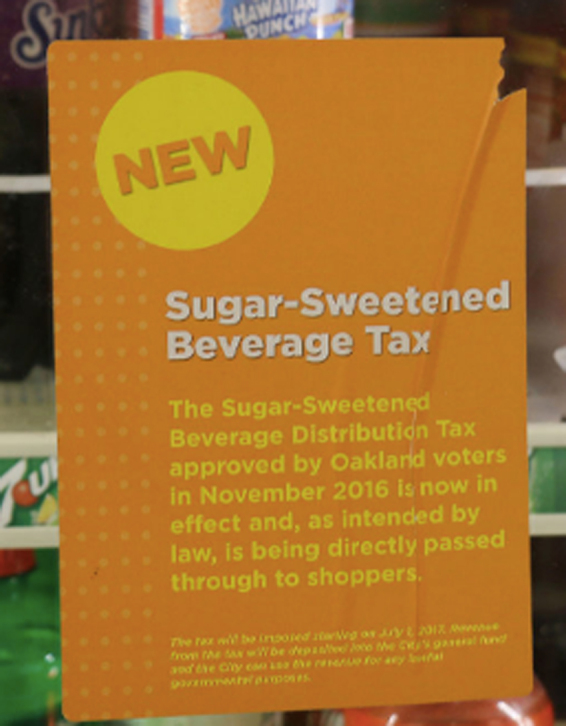
Orange Signs (Source: Sugar Freedom Project).

Shortly after tax passage, stakeholders recalled mysterious “orange signs” displayed in local grocery stores sharing inaccurate messaging (Fig. 1). While the signs had no branding or logo, and no one could pinpoint origin, the content framing clearly opposed the tax.^44^ The Sugar Freedom Project documented the disproportionate prevalence of the orange signs, finding more signage in lower-income areas (e.g., Flats neighborhood, 73% of signs) compared to more affluent areas (e.g., Hills neighborhood, 7% of signs).^34^

## Discussion

This longitudinal case study highlights the importance of ordinance language for bolstering the underlying “spirit” of equity, particularly in CAB composition requirements and responsibilities all with the purpose of engaging and representing communities marginalized by structural inequities.^45^ In the seven U.S. jurisdictions with effective beverage taxes, five include language for community representative advisory boards and/or in guidance for revenue spending to address equity; for example, San Francisco and Berkeley both have advisory committees that advocate for health equity in communities disproportionately impacted by SSBs.^26,46^ Notably, such explicit policies provide a blueprint for the intent of tax implementation and evaluation. In Oakland, a future planned tax evaluation and impact study will rely on the Department of Race and Equity's involvement and will be conducted by a third-party evaluator;^34^ which is important for conducting an impartial and equitable evaluation.

A critical strategy for ensuring equitable funding recommendations is in the appointing of an independent CAB, which, in Oakland, represented the diverse community both in composition and policy implementation, through leadership and partnerships. Engaging local advocacy organizations with existing ties to communities marginalized by structural inequities afforded the CAB, and thus the City, an intimate knowledge of the cultural and historical injustices leading to disparities (e.g., OUSD elevated lead levels).^45^ The literature on equitable implementation of SSB taxes is limited, but broader literature emphasizes inclusivity, building trust, and engaging community members, particularly those marginalized communities, to partake in implementation decisions.^27–29^ Jurisdictions interested in equitable implementation should consider their current capacity to access and engage community members. Identifying partnerships and establishing ways to increase capacity for engaging communities will facilitate sustainable policy implementation.

The beverage industry launched an anti-tax campaign, consistent with their opposition campaign to Berkeley's tax,^26^ claiming an SSB tax would lead to a full-scale “grocery tax.” Also consistent with other jurisdictions was the industry's targeting of minority communities^3–6,47^ through a sympathetic appeal for community-faced economic insecurities. Contrary to grocery tax claims that would increase hardships in lower income communities, Berkeley documented no increase in food prices after implementation of the SSB tax.^26^ The Oakland CAB's coordinated partnerships and outreach responded to industry attacks in lower income communities of color, providing a mechanism to counter the opposition's claims. Policymakers and advocates in other jurisdictions should prepare counter-strategies for targeted campaign attacks both before adoption and during implementation.

This study has several limitations. Themes are derived from implementation stakeholder accounts, triangulated with documentation, but not gleaned from observed or objective measures of implementation activities. In addition, anti-tax stakeholder accounts (e.g., beverage industry representatives) and residents from lower income communities of color were not directly included in the KI sample. Industry perspectives were captured through media articles and community perspectives were captured through local advocacy groups and media articles.

## Conclusion

Measure HH serves as an example for how policymakers incorporated equity through ordinance language. The Oakland CAB offers an example for liaising between the City and community partners and leveraging community networks to create implementation mechanisms providing access to marginalized residents and local inequities. Partners and community access afforded the CAB the ability to counter strong and ongoing attacks from the beverage industry's anti-tax campaign. The additional Measure HH requirement to “establish/and/or fund programs to prevent or reduce the consequences of sugar-sweetened beverages on health” in communities “most affected by health disparities”^31^ provided steps for dedicated funding beyond the initial implementation phases. In concert with the CAB and its extended network, this funding was able to provide support to residents in communities marginalized by structural inequities.

## Abbreviations Used

CAB: Community Advisory Board
KI: key informant
OUSD: Oakland Unified School District
SES: socioeconomic status
SSB: sugar-sweetened beverage
